# Identification of iron metabolism-related genes in the circulation and myocardium of patients with sepsis *via* applied bioinformatics analysis

**DOI:** 10.3389/fcvm.2023.1018422

**Published:** 2023-03-02

**Authors:** Renlingzi Zhang, Chong Di, Hanlu Gao, Yunlou Zhu, Congye Li, Zhengfang Zhu, Qixing Wang, Junjie Wang, Feng Zhou, Sheng Wang

**Affiliations:** Department of Critical Care Medicine, Shanghai Tenth People’s Hospital, School of Medicine, Tongji University, Shanghai, China

**Keywords:** sepsis, septic cardiomyopathy, iron metabolism, peripheral blood monocytes, diagnostic biomarkers

## Abstract

**Background:**

Early diagnosis of septic cardiomyopathy is essential to reduce the mortality rate of sepsis. Previous studies indicated that iron metabolism plays a vital role in sepsis-induced cardiomyopathy. Here, we aimed to identify shared iron metabolism-related genes (IMRGs) in the myocardium and blood monocytes of patients with sepsis and to determine their prognostic signature.

**Methods:**

First, an applied bioinformatics-based analysis was conducted to identify shared IMRGs differentially expressed in the myocardium and peripheral blood monocytes of patients with sepsis. Second, Cytoscape was used to construct a protein–protein interaction network, and immune infiltration of the septic myocardium was assessed using single-sample gene set enrichment analysis. In addition, a prognostic prediction model for IMRGs was established by Cox regression analysis. Finally, the expression of key mRNAs in the myocardium of mice with sepsis was verified using quantitative polymerase chain reaction analysis.

**Results:**

We screened common differentially expressed genes in septic myocardium and blood monocytes and identified 14 that were related to iron metabolism. We found that *HBB*, *SLC25A37*, *SLC11A1*, and *HMOX1* strongly correlated with monocytes and neutrophils, whereas *HMOX1* and *SLC11A1* strongly correlated with macrophages. We then established a prognostic model (*HIF1A* and *SLC25A37*) using the common differentially expressed IMRGs. The prognostic model we established was expected to better aid in diagnosing septic cardiomyopathy. Moreover, we verified these genes using datasets and experiments and found a significant difference between the sepsis and control groups.

**Conclusion:**

Common differential expression of IMRGs was identified in blood monocytes and myocardium between sepsis and control groups, among which *HIF1A* and *SLC25A37* might predict prognosis in septic cardiomyopathy. The study may help us deeply understand the molecular mechanisms of iron metabolism and aid in the diagnosis and treatment of septic cardiomyopathy.

## 1. Introduction

Sepsis is a life-threatening clinical syndrome caused by a dysfunctional host response to an infection (1). It is the leading cause of death in patients who are critically ill, and its mortality significantly increases when combined with multiple dysfunctions, leading to functional failure of essential organs, such as the heart, liver, lungs, and kidneys (2). The fatality of sepsis is attributed to organ interdependence; that is, failure of one organ often leads to dysfunction or failure of other organs. The interdependence is particularly evident in cardiovascular failure, which reduces overall blood circulation, exacerbating mitochondrial dysfunction, tissue hypoxia, and metabolic imbalance (3). Immunosuppression in late sepsis contributes to progressive cardiovascular failure, which is often the last step before sepsis-induced death (4).

Iron metabolism plays a vital role in sepsis. High plasma ferritin levels can be identified as “macrophage activation-like syndrome” in patients with sepsis. Hyperferritinemia has been evaluated as a new inflammatory response biomarker that may provide a new strategy for the clinical study of anti-inflammatory effects (5). Lipid peroxidation is stimulated when large quantities of iron are released from necrotic tissue and damaged mitochondria, and hydroxyl radicals, the most effective reactive oxygen species, are produced (6). This chain reaction accelerates cell membrane damage, ultimately leading to cell death. Research has shown that iron metabolism imbalance is common in many cardiovascular diseases. Nutrient-related iron deficiency affects 75% of patients with heart failure (7). Both primary and secondary iron overload can induce heart disease through oxidative stress, but the specific mechanisms remain unknown (8–10). Excess iron in myocardial cells can also induce ferroptosis through the accumulation of peroxides in the cell membrane (9). Iron metabolism disorders play vital roles in the pathogenesis of septic cardiomyopathy (11, 12). Therefore, further understanding of iron disorders in septic cardiomyopathy would help discover effective targets for therapeutic intervention.

The pathological mechanism of septic cardiomyopathy has not been fully elucidated (13). Transcriptome sequencing in sepsis research has enabled us to gain an understanding of the differentially expressed genes (DEGs), how they are involved in sepsis, and the impact of signaling pathways. Previous studies have focused on transcriptomic differences in peripheral blood, but functions of the screened candidate genes could not be correlated with diseases involving multiple organ damage due to a large number of DEGs. Iron overload exists in septic cardiomyopathy; however, the regulatory genes of iron metabolism involved in the development of sepsis are unknown. Circulating immune cells are the link between local and systemic organ inflammation. To the best of our knowledge, this is the first integrated study to focus on iron metabolism-related genes (IMRGs) from transcriptomic data of septic cardiomyopathy and blood monocytes.

In this study, we performed applied bioinformatics analysis and screened for differentially expressed IMRGs in blood monocytes and heart samples of patients with sepsis. A prognostic model of IMRGs was established based on DEGs shared by septic myocardium and peripheral blood. Based on these candidate genes, we predicted targeted small-molecule drugs. Ultimately, this study may assist clinical diagnosis and treatment of septic cardiomyopathy.

## 2. Materials and methods

### 2.1. Differential expression analysis of iron metabolism-related genes

We obtained clinical information from the Gene Expression Omnibus (GEO) database and datasets stored by Matkovich SJ (14) (GSE79962) and Biswas SK (15) (GSE46955) were downloaded. The cardiac tissue samples from 20 patients with septic cardiomyopathy and 11 patients without heart failure were included in the GSE79962 dataset. Blood monocyte samples in the GSE46955 dataset were collected from patients with gram-negative sepsis during infection and post-recovery, as well as from healthy donors. Raw data from GSE769962 and GSE65682 datasets were preprocessed using the R “affy” package (16) (version 3.8; R Foundation for Statistical Computing, Vienna, Austria), and raw data from the GSE46955 dataset were preprocessed using the R “limma” package (17) (version 3.42.2). A total of 507 IMRGs were obtained from the Molecular Signatures Database (MSigDB) version 7.4 (18).

The mRNA expression profiles of sepsis and control samples, including cardiac tissues and blood monocytes, were compared to identify DEGs with (|FC|) >1.5 and adjusted *p* < 0.05. Volcano plots and heatmaps of IMRGs were constructed using the “ggplot2” package (version 3.3.3) and R (version 3.6.3) (19). A *t*-test was used to determine *p*-values and adjusted *p*-values in the differential gene expression analysis. We identified the common IMRGs *via* an Online Venn diagram tool.^1^

### 2.2. Functional enrichment analysis

The R “clusterProfiler” (version 3.14.3) package was used to visualize enrichment analysis and biological processes (20). To investigate potential biological functions between sepsis and controls, Gene ontology (GO), and Kyoto Encyclopedia of Genes and Genomes (KEGG) pathway enrichment analyses were conducted using R package “clusterProfiler” (21). Differences at *p* < 0.05 were considered statistically significant.

### 2.3. Protein–protein interaction network analysis

The STRING online database^2^ was used to assess functional relatedness and can be applied for protein–protein interactions (PPI) network analysis (22). Cytoscape (version 3.6.0) was used to visualize the network, with interaction scores set at >0.4. Genes and the relationship between genes are represented by nodes and edges, respectively. To identify key PPI network modules, molecular complex detection (MCODE) was used for gene network clustering analysis (23). CytoHubba in Cytoscape (version 0.3) was used to estimate essential nodes in the PPI network *via* different topological analysis methods (24).

### 2.4. Immune microenvironment and prognostic prediction model

To assess the immune response in sepsis, the single-sample gene set enrichment analysis (ssGSEA) algorithm was applied to estimate immune infiltration for each sample in the GSE79962 dataset (25). Additionally, a prognostic prediction model for IMRGs was constructed using a multivariate Cox regression analysis based on the univariate Cox regression analysis, and the patient’s risk scores were calculated for the GSE65682 dataset. Kaplan–Meier survival curve analyses were compared between high- and low-risk groups, and receiver operating characteristic (ROC) curves were used to evaluate the model (26).

### 2.5. Drug prediction of iron metabolism-related genes

The Drug Signatures Database is a collection of drug- and small-molecule-related gene sets. We searched for potential IMRGs in this database, and 10 predicted drugs are listed in Table 1 according to the combined score (27).

**Table 1:** Targeted drug prediction.

Index	Term	*P*-value	Odds ratio	Combined score	Genes
1	Allococaine CTD 00005697	2.11E-04	166.013966	3795.72462	*SLC11A1; HMOX1; HBB; HAMP; SLC39A14; HIF1A*
2	Amcinonide TTD 00001949	0.0010997	332.5	6410.49953	*HMOX1; HBB; HAMP; HIF1A*
3	Benzamil CTD 00000704	0.0010997	309.255814	5878.1503	*MT2A; HMOX1; HBB; HIF1A*
4	Razoxane CTD 00006692	0.0010997	112.579545	2036.30404	*MT2A; HMOX1; SOD2; HAMP; HIF1A*
5	L-mimosine CTD 00006335	0.0010997	237.309524	4272.70515	*MT2A; HMOX1; SOD2; HIF1A*
6	Oxatomide TTD 00009969	0.00119964	108.835165	1950.77096	*HMOX1; HBB; SOD2; HAMP; HIF1A*
7	Beclomethasone TTD 00002323	0.00119964	856.285714	14966.0259	*HMOX1; HAMP; HIF1A*
8	Levonordefrin TTD 00008958	0.00119964	170.188034	2847.71939	*SLC11A1; HMOX1; HBB; SOD2*
9	Oxatomide CTD 00000800	0.00119964	56.2745665	934.800854	*MT2A; HMOX1; HBB; SOD2; HAMP; HIF1A*
10	FERRIC CITRATE CTD 00001186	0.00119964	63.2765273	968.503885	*MT2A; HMOX1; SOD2; SLC39A14; HIF1A*

The top 10 drugs were predicted using the Drug Signatures Database based on common iron metabolism-related genes.

### 2.6. Lipopolysaccharide-induced septic cardiomyopathy In mice

Twenty healthy C57BL/6 male mice between 8 and 10 weeks of age were provided by Shanghai Jisijie Laboratory Animal Co., Ltd. (Shanghai, China), and all experiments were performed at the Experimental Center of Tongji University (Shanghai, China) in a constant temperature and humidity environment. All mice in this study were raised at a regular circadian rhythm with free access to food and water. All animal procedures followed the Guide for the Care and Use of Laboratory Animals published by the National Institutes of Health (Bethesda, MD, USA) in 1996, and all animal research was approved by the Animal Research Ethics Committee of Shanghai Tenth People’s Hospital (Shanghai, China).

Lipopolysaccharide (10 mg/kg) was administered to mice intraperitoneally. After 12 h, the mice were examined using transthoracic echocardiography and then euthanized with carbon dioxide anesthesia. Heart tissue was collected, fixed in 4% paraformaldehyde, and embedded in paraffin for histological analysis. According to standard technical protocols, the heart samples were sliced into sections of 4–5 mm and then stained with hematoxylin and eosin (H&E). All photomicrographs were acquired using a light microscope (TE2000-U; Nikon, Tokyo, Japan) at 200× magnification.

The left ventricular function of mice was examined using transthoracic echocardiography with an ultrasound system (Vevo 2,100; Vision Electronics, Toronto, ON, Canada) under isoflurane anesthesia. Accordingly, the left ventricular ejection fraction (LVEF) and fractional shortening (FS) were calculated. All measurements used for analysis were the average of three consecutive cardiac cycles.

Mice were euthanized after 12 h, and the total RNA in the myocardium was isolated using TRIzol reagent (Invitrogen, Carlsbad, CA, USA) according to the manufacturer’s instructions. A Nanodrop Nd1000 analyzer (Thermo Fisher Scientific, Waltham, MA, USA) was used to measure the quantity and purity of the total RNA. According to the manufacturer’s instructions, a Hieff 1st Strand cDNA Synthesis Kit (11121ES60; Yeasen, Shanghai, China) was used to reverse transcribe total RNA into cDNA. The Hieff SYBR Green Master Mix qPCR Kit (low Rox plus 11202es08; Yeasen) was used to perform quantitative polymerase chain reaction (qPCR) on a real-time PCR detector (QuanStudio; Applied Biosystems, Waltham, MA, USA). The primer pairs used are shown in Supplementary Table 1. Values were normalized to those of β-actin using the 2^−ΔΔCt^ method.

### 2.7. Identifying iron metabolism-related genes associated with cardiovascular and inflammatory diseases

We analyzed the relationship between IMRGs and cardiovascular and inflammatory diseases using the comparative toxicogenomics (CTD) database, and the inference scores (the degree to which iron metabolism genes are related to the disease) were determined.

### 2.8. Statistical analysis

Student’s *t*-test was used to compare continuous variables, and Chi-square or Fisher’s exact tests were used to compare categorical variables. The diagnostic accuracy of the two hub genes was analyzed using the survival curve analysis and expressed by the area under the ROC curve at a 95% confidence interval. Spearman’s rank test or Pearson correlation coefficient was used to analyze the correlation between potential genes and immune cells. All statistical analyses were performed using R software (version 3.6.3) and SPSS (version 19.0; SPSS Inc., Chicago, IL, USA). *p*-values less than 0.05 (bilateral) were considered statistically significant.

## 3. Results

### 3.1. Differentially expressed iron metabolism-related genes in septic cardiomyopathy

**Figure 1:**
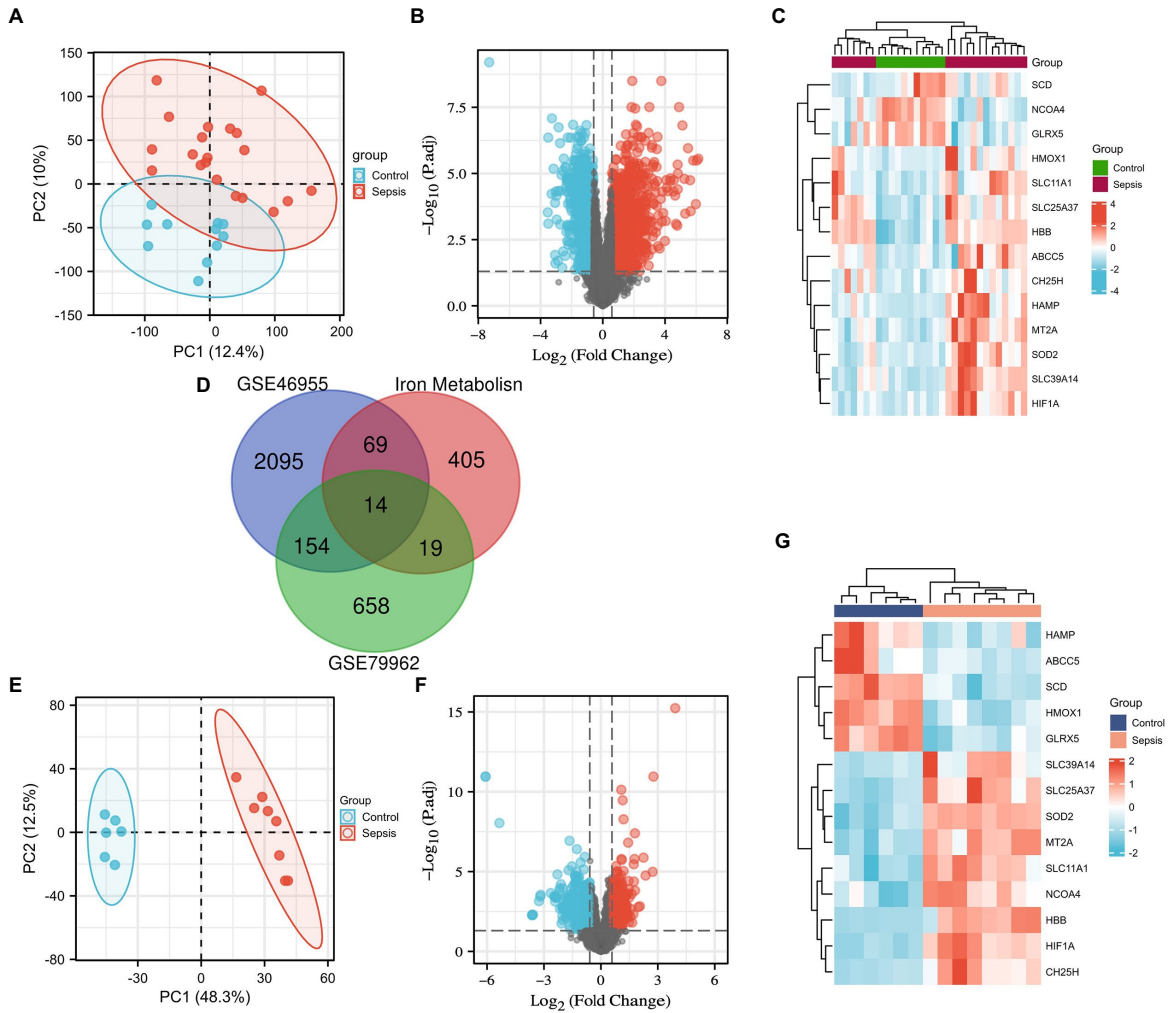
Screening of the differentially expressed iron metabolism-related genes (IMRGs). **(A)** Principal components analysis for GSE79962. **(B)** Volcano plot of GSE79962. **(C)** Heatmap of the common differentially expressed IMRGs in septic myocardiopathy and controls. **(D)** Venn diagram showing the overlap of genes among differentially expressed genes (DEGs) of GSE79962, GSE46955, and IMRGs. **(E)** Principal components analysis of GSE46955. **(F)** Volcano plot of GSE46955. **(G)** Heatmap of the common differentially expressed IMRGs in blood monocytes of sepsis and control groups.

We downloaded the microarray RNA expression profiling datasets, GSE79962 and GSE46955, from the GEO database. In total, we identified 83 and 33 IMRGs differentially expressed in peripheral blood monocytes and myocardial tissues, respectively. Principal component analysis (PCA) was performed, which revealed that patients in GSE79962 were well separated in the two major dimensions (Figure 1A). Expression distribution across all genes between the non-heart failure and septic myocardium groups is depicted in a volcano plot (Figure 1B), and the common IMRGs are presented in a heatmap (Figure 1C). We identified 14 common DEGs upon comparing the iron metabolism-related DEGs between septic myocardial tissue and blood monocytes by overlapping the Venn diagrams (Figure 1D). PCA of the GSE46695 dataset indicated the relatedness of sample transcriptomes when comparing sepsis with non-sepsis groups (Figure 1E). Following analysis of the GSE46955 dataset, the DEGs between the control and sepsis groups were presented as a volcano plot (Figure 1F), and the common iron metabolism-related DEGs in a heatmap (Figure 1G).

### 3.2. Go and KEGG enrichment analysis

**Figure 2:**
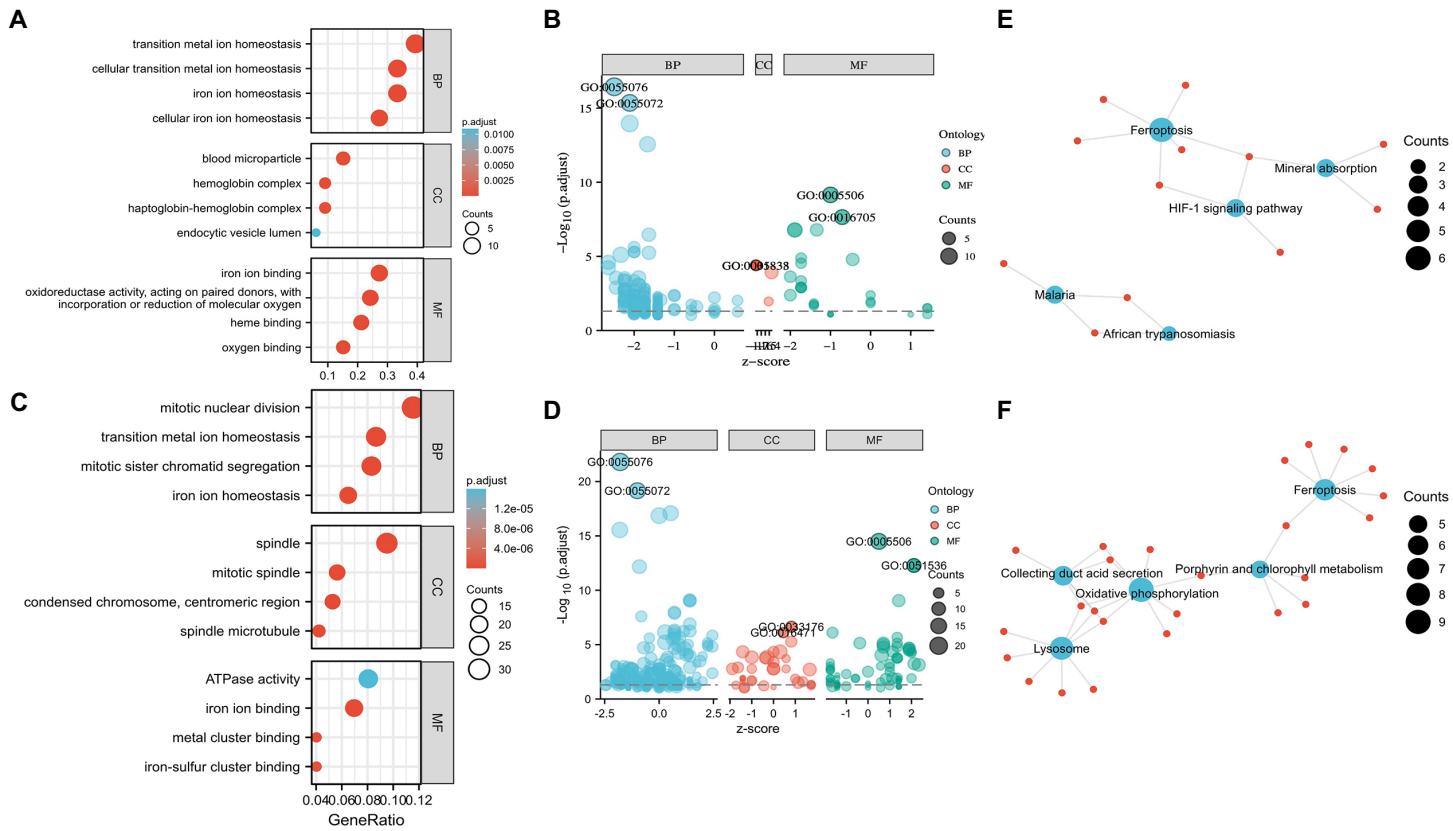
Enrichment analysis for the differentially expressed genes (DEGs) in circulation and the myocardium. **(A)** Gene Ontology (GO) enrichment analysis, **(B)** Bubble plot of enriched GO terms in GSE79962. **(C)** GO enrichment analysis, **(D)** Bubble plot of enriched GO terms in GSE46955. **(E)** Kyoto Encyclopedia of Genes and Genomes (KEGG) pathway analysis in GSE79962. **(F)** KEGG pathway analysis in GSE46955. Abbreviations: BP, biological process; CC, cellular component; MF, molecular function.

Functional enrichment of the DEGs enabled the recognition of the main similarities and differences in the expression profiles of the IMRGs in myocardial samples and blood monocytes of patients with sepsis. GO and KEGG analyses were applied to identify the activated iron metabolic signaling pathways in the myocardium and blood monocytes of patients with sepsis. GSE79962 dataset analysis revealed that the most significantly enriched GO terms involved cellular transition metal ion homeostasis, blood microparticle, and iron ion binding (Figures 2A,B). Furthermore, GSE46955 dataset analysis revealed that the most significantly enriched GO terms involved mitotic nuclear division, spindle, and iron ion binding (Figures 2C,D). Most importantly, KEGG enrichment analysis revealed that the differentially expressed IMRGs from both datasets were mainly involved in the process of ferroptosis (Figures 2E,F).

### 3.3. Protein–protein interaction network

**Figure 3:**
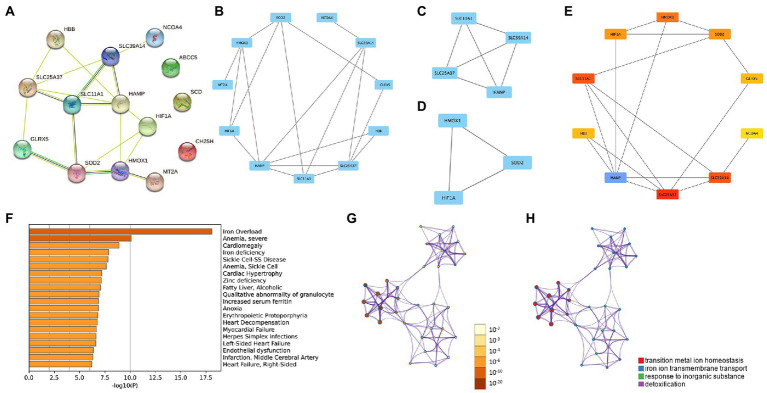
Protein–protein interaction (PPI) networks in common DEGs of iron metabolism. **(A)** PPI network formed by common differentially expressed IMRGs. **(B–D)** The top three MCODE complexes. **(E)** The top 10 hub genes identified by Cytohubba. **(F–H)** Enrichment analysis performed by Metascape.

The PPI network enabled the investigation of the molecular mechanisms of diseases and the identification of potential IMRGs. We performed a PPI analysis of 14 common IMRGs in blood monocytes and myocardium of patients with sepsis (Figure 3A). The MCODE plugin in Cytoscape revealed highly interconnected modules in the PPI network and a total of three functional modules were identified (Figures 3B–D). The top 10 hub genes were screened using the maximal clique centrality method with CytoHubba plugin (Figure 3E). These included *HAMP*, *SLC25A37*, *SLC11A1*, *SLC39A14*, *HMOX1*, *SOD2*, *HIF1A*, *GLRX5*, *HBB*, and *NCOA4*. The scores of hub genes identified by Cytohubba are shown in Supplementary Table 2. Using Metascape enrichment analysis, we established that these genes were related to iron overload, cardiomegaly, and transition metal ion homeostasis (Figures 3F–H).

### 3.4. Immune cell infiltration of heart samples

**Figure 4:**
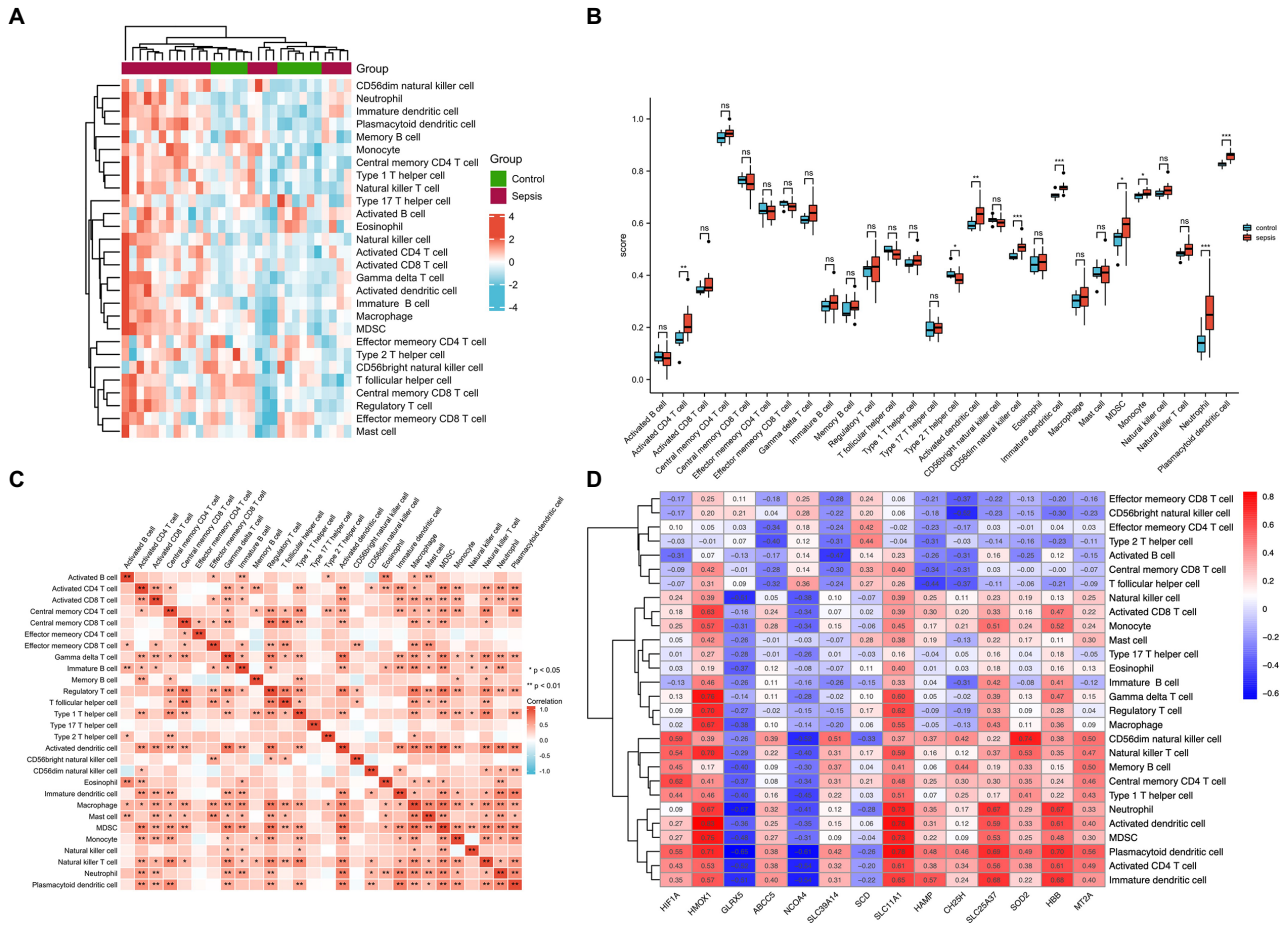
Landscape of immune infiltration in the myocardium of patients with sepsis and controls. **(A)** Heatmap of the proportions of the 28 immune cell types. **(B)** Box plots of immune cell proportions. **(C)** Correlation analysis of immune cells. **(D)** Correlation between common differentially expressed IMRGs and immune cells.

To identify immune cell signatures in septic myocardium, the ssGSEA algorithm was used to analyze the proportion of immune cell infiltration. A heatmap depicting the proportions of immune cell types was constructed (Figure 4A). Box plots of 28 immune cells in sepsis samples (Figure 4B) and their correlations with immune cells were plotted (Figure 4C). For innate cells, neutrophils, monocytes, as well as activated dendritic, CD56 bright natural killer, immature dendritic, and plasmacytoid dendritic cells indicated significant differences between the septic myocardium and control groups. For adaptive immune cells, myeloid-derived suppressor, activated CD4 T, and type 2 T helper cells indicated significant differences. Correlation analysis between immune cells revealed that 15 immune cells were significantly correlated with monocytes. We evaluated the association between IMRGs and immune infiltration characterization in septic myocardium. Strong correlations were also observed between potential IMRGs (*HMOX1*, *SLC11A1*, *SLC25A37*, and *HBB*) and monocytes and neutrophils, whereas *HMOX1* and *SLC11A1* with macrophages. A negative correlation was observed between monocytes and *GLRX5* and *NCOA4* expression (Figure 4D). These results further support the notion that IMRGs may affect immune cell activity.

### 3.5. Prognostic prediction model

**Figure 5:**
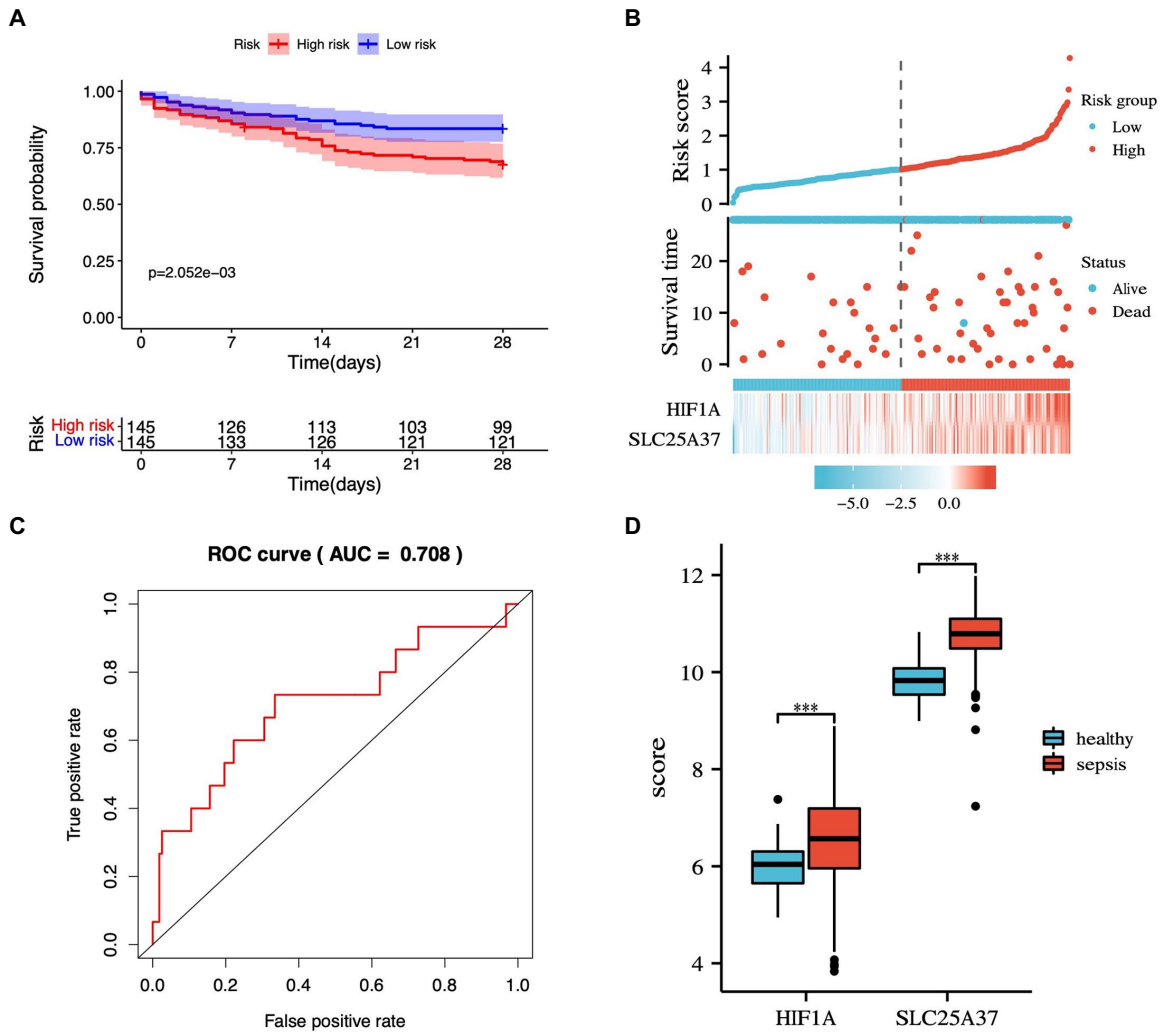
Prognostic prediction model based on common IMRGs. **(A)** Kaplan–Meier survival curves of 28-day mortality between high- and low-risk groups (*p* = 2.052e-03). **(B)** The risk score analysis between high-risk and low-risk groups and survival status analysis. **(C)** The receiver operating characteristic (ROC) curve of the two-gene model in patients with sepsis and controls (AUC:0.708). **(D)** The expression of the two genes between sepsis and healthy samples.

The potential IMRGs obtained by univariate Cox regression analysis were further incorporated into multivariate Cox regression analysis. This led to the identification of two iron metabolism genes related to sepsis prognosis (*HIF1A* and *SLC25A37*). The expression value and relative coefficients of two genes were used to calculate the survival risk score (Supplementary Table 3). The cutoff value of a patient’s risk score was set at 1.007. Based on the median risk score value, sepsis samples in GSE65682 were divided into high- and low-risk groups. After multivariate regression analysis, we found no statistically significant differences in age, gender, and diabetes except for risk score (Supplementary Figure 1). Using survival curve analysis, we observed a significant difference between the high- and low-risk group models (*p* = 2.052e-03; Figure 5A). Survival analysis revealed that two iron metabolism-related DEGs were associated with sepsis prognosis (Figure 5B). An ROC curve was plotted, and the area under the ROC curve for the experimental dataset GSE65682 was 0.708 (Figure 5C). We used the GSE65682 dataset to identify the expression of these two genes between sepsis and non-sepsis samples and found that their expressions changed significantly (Figure 5D).

### 3.6. Validation in septic myocardium in mice using quantitative PCR analysis

**Figure 6:**
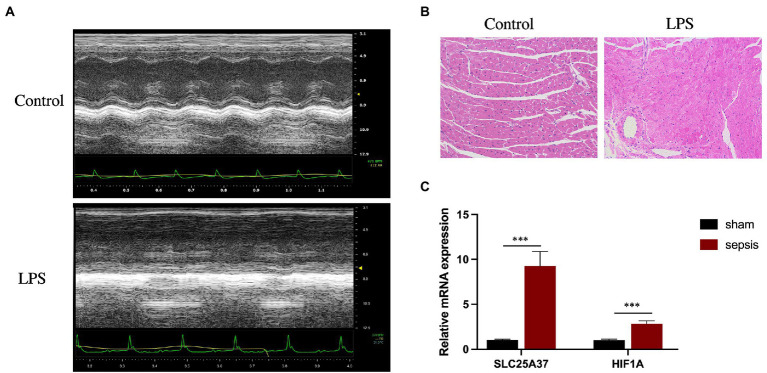
Validation of *SLC25A37* and *HIF1A* in a mouse model of LPS-induced sepsis using quantitative PCR analysis. **(A)** Transthoracic echocardiography between sepsis and control groups. **(B)** Hematoxylin and eosin staining. **(C)** Relative expression of *SLC25A37* and *HIF1A* between sepsis and control groups in mice, along with the expression of β-actin as an internal standard for normalization. Asterisks indicate a significant statistical *p*-value calculated using Student’s *t*-test (*p < 0.05; **p < 0.01; ***p < 0.001).

Echocardiography revealed that, compared with those in the control group, LPS treatment significantly reduced the LVEF (%) and FS (%) of the mice (Figure 6A). Furthermore, H&E staining revealed cardiomyocyte disarray in the LPS group compared with that of the control. (Figure 6B). To verify the reliability of the two IMRGs (*HIF1A* and *SLC25A37*), we evaluated the mRNA expression of key genes in the myocardium of mice. As shown in Figure 6C, the expressions of *HIF1A* and *SLC25A37* were significantly higher in the sepsis group compared with that in the control group. The expression levels (logFC) of key genes with values from microarray and qPCR for comparison are listed in Supplementary Table 4.

### 3.7. Iron metabolism-related genes and diseases

**Figure 7:**
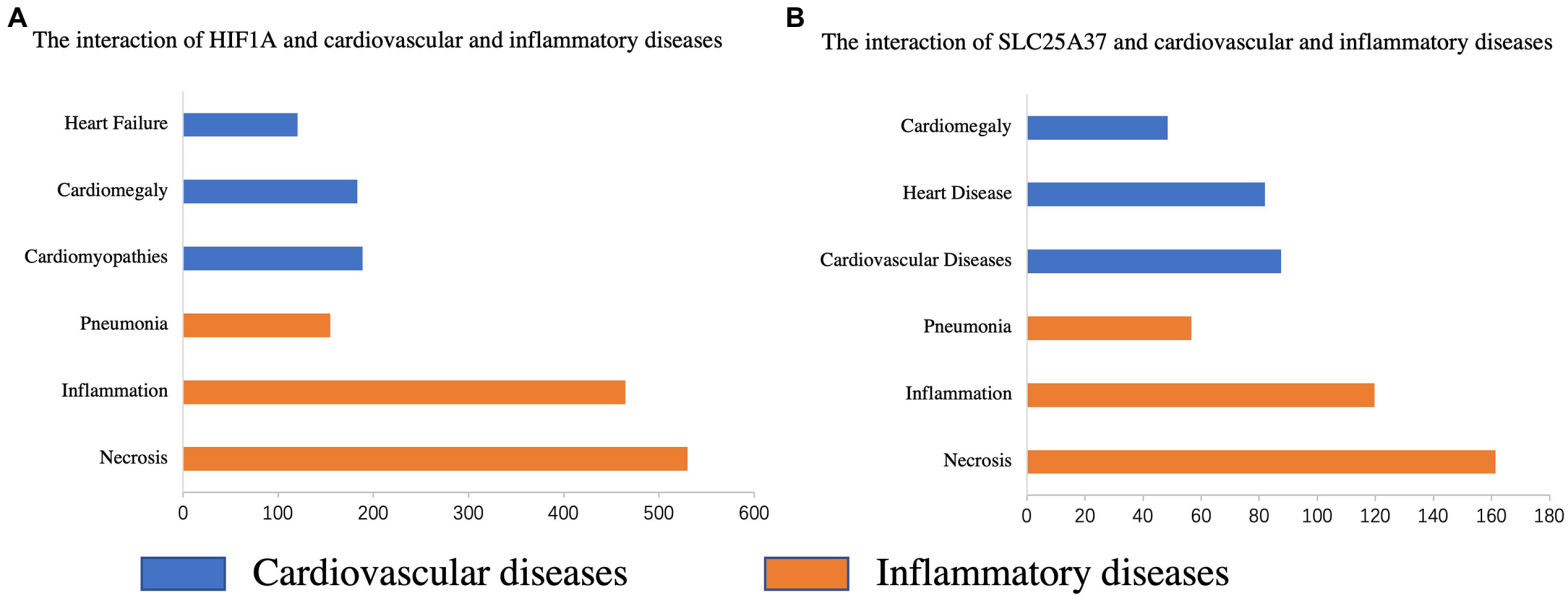
Relationship between the genes of the prognostic model and various diseases based on the comparative toxicogenomics database (CTD). Interactions between *HIF1A* **(A)** and *SLC25A37* **(B)** and cardiovascular and inflammatory diseases, respectively.

The CTD database was used to search for two common genes related to cardiovascular and inflammatory diseases. *HIF1A* was significantly associated with pneumonia (Inference Score: 161.07), inflammation (Inference Score: 467.34), and necrosis (Inference Score: 536.32), and was highly associated with certain cardiovascular diseases, including heart failure (Inference Score: 118.29), cardiomegaly (Inference Score: 190.62), and cardiomyopathies (Inference Score: 200.80) (Figure 7A). Similarly, *SLC25A37* was associated with pneumonia (Inference Score: 56.82), inflammation (Inference Score: 119.76), and necrosis (Inference Score: 161.39), and was highly associated with cardiovascular diseases (Inference Score: 87.62), heart diseases (Inference Score: 82.00) and cardiomegaly (Inference Score: 48.33) (Figure 7B).

## 4. Discussion

Sepsis-induced cardiomyopathy or sepsis-induced myocardial dysfunction is increasingly recognized as a transient form of cardiac insufficiency during sepsis, with a notable impact on the prognosis of patients (28). Among the many pathogenic factors of septic cardiomyopathy, there is growing concern regarding the critical role that iron metabolism disorder plays in septic cardiomyopathy. Among the DEGs in septic cardiomyopathy tissue and peripheral blood monocytes, we found shared IMRGs and evaluated their correlation with the immune cells of septic cardiomyopathy. We established a prognostic model (*HIF1A*, *SLC25A37*) using the common differentially expressed IMRGs of septic myocardium and blood monocytes. The prognostic model we established might assist in the diagnosis of septic cardiomyopathy. We verified the potential genes in the myocardium of mice and found a significant difference between the sepsis and control groups.

Monocyte recruitment occurs after tissue damage caused by various infectious and aseptic inflammations. In cardiovascular disease, mobilization and infiltration of peripheral blood monocytes into diseased tissues are mainly adverse adaptation reactions (29). In a steady state, the heart of an adult mammal contains two macrophage populations: one from embryonic development and the other from blood monocytes (30). Under cardiac stress, monocyte-derived macrophages express high levels of pro-inflammatory genes, and their inappropriate activation in a sterile inflammatory environment may lead to pathological processes in the body (31). Recent advances in septic transcriptomics research have contributed to a better understanding of gene expression and its typical signaling pathways. Substantial evidence suggests significant changes in the leukocyte transcriptome in patients with sepsis; therefore, different patient phenotypes become apparent. Leukocytes of critically ill patients demonstrate profound changes in the transcriptome, in which 70–80% of RNA transcripts are differentially expressed compared with those in healthy individuals (32). The relationship between peripheral blood monocytes and cardiomyopathy in sepsis has not been clarified. Investigating potential IMRGs in peripheral blood monocytes and septic myocardium may provide a new approach to treating septic cardiomyopathy.

Sepsis can result in life-threatening organ dysfunction caused by the dysregulated host response to an underlying infection and is characterized by unbalanced inflammation and immunosuppression (32). The role of immunity in the pathogenesis of septic cardiomyopathy is self-evident. We used ssGSEA to analyze the immune infiltration of sequencing data in septic myocardium. Our research showed that innate immune cells, including neutrophils and monocytes, were significantly upregulated. Iron has multiple effects on the immune system, including regulating immune cell proliferation and differentiation, controlling various effector mechanisms of immune cells, and directly interfering with antimicrobial immune effector pathways. We analyzed the correlation between common IMRGs and immune cells of septic myocardium. Our study found that *HBB*, *SLC25A37*, *SLC11A1*, and *HMOX1* have a strong correlation with monocytes and neutrophils, and *HMOX1* and *SLC11A1* have a strong correlation with macrophages. The incidence of sepsis-associated anemia is second only to iron deficiency anemia and is classified as a chronic anemic disease. When the expression of erythrocyte membrane proteins is altered, and erythrocyte deformability is reduced, hemolysis is more likely to occur during sepsis (33). Heme may increase host susceptibility to infection by inducing heme oxygenase 1 (HMOX-1) in immature neutrophils, thereby inhibiting the oxidative burst required to clear phagocytic bacteria (34). Hemolysis may also favor anti-inflammatory, immune cell polarization by inhibiting the dendritic cell maturation that is required for effector T cell responses. This may then induce monocyte differentiation into red plasma macrophages and drive regulatory T-cell expansion by regulating HMOX-1 expression in non-classical monocytes (35).

Inflammation causes changes in the genes related to iron metabolism. Ligands for Toll-like receptors 2, 4, and 6, BMP2, BMP6, α1 antitrypsin, activin B, IL-1, and IL-22 are examples of inflammation-driven hepcidin inducers (36–39). However, not all iron metabolism genes are equally regulated in monocytes and the myocardium. For example, hepcidin, known as hepcidin antimicrobial peptide (HAMP), is a 25-amino acid protein mainly produced by hepatocytes, but its expression and release are also detected in other organs, including the heart (40). When the iron levels in the body are high, hepcidin can promote iron-exporter ferroportin degradation and thus inhibit ferritin efflux. Hepcidin can also be released under conditions such as iron overload, red blood cell transfusion, iron therapy, or inflammation (36). We found that the mRNA expression level of *HAMP* is upregulated in the myocardium but downregulated in monocytes. When systemic hepcidin concentrations are significantly low, hepcidin produced by the myocardium acts as an autocrine or paracrine regulator of ferroportin and can protect tissues from ferroportin and extreme iron deficiency; however, this does not significantly affect systemic hepcidin (40).

We identified two IMRGs using the Cox regression method to establish a prognostic model (*HIF1A* and *SLC25A37*). The ROC curve shows that this model may have excellent diagnostic ability for sepsis. Juan José Martínez-García found that hypoxia-inducible factor (HIF)-1 α is activated, possibly through the influence of its P2X7 receptor on HIF-1 α, and that inhibition of NLRP3 leads to mitochondrial damage in the monocytes of patients with sepsis (41). Functional plasticity or reprogramming occurs in circulating monocytes during sepsis, and the expression and activity of *HIF-1α* are upregulated (15), which is consistent with our results. Studies have shown that *SLC25A37* is related to mitochondrial iron accumulation in pancreatic cancer. *SLC25A37* mediated by the *PINK1*-*PARK2* pathway increases mitochondrial iron accumulation, which leads to the *HIF1A*-dependent Warburg effect and *AIM2*-dependent inflammasome activation in tumor cells (42).

There is a lack of effective treatment to regulate the complex pathophysiology of the host immune response. Traditional treatment of sepsis includes timely implementation of supportive treatment, such as fluid resuscitation, vasopressor and oxygen therapy, mechanical ventilation, timely source control, and medicines such as antibiotics (43). Previous clinical trials have partly focused on anti-inflammatory therapy, but were unsuccessful in combating the inflammatory storm of sepsis (43). Iron chelation has immunomodulatory effects and can improve the prognosis of sepsis models (44, 45). Therefore, iron chelators may become new targets for developing anti-sepsis medicine. In the present study, we predicted small-molecule drugs based on differential IMRGs, and studies have shown that some drugs have therapeutic effects on heart diseases in basic experiments (46–49).

Due to limited research data, this study presented some limitations. First, although we validated potential iron metabolism genes in a mouse model of septic cardiomyopathy, this was lacking in the peripheral blood of those with septic cardiomyopathy. Therefore, we require clinical randomized controlled studies on sepsis-induced cardiomyopathy for further validation. Second, the proportion of immune cells was inferred using the ssGSEA algorithm rather than a real test of sepsis cardiomyocytes, which may show differences from the actual results. Third, the specific regulatory mechanism of iron metabolism genes in septic cardiomyopathy found in this study has not been deeply elucidated and further exploration is required.

## 5. Conclusion

In conclusion, we identified the shared differential expression of IMRGs in myocardium tissue and blood monocytes and analyzed the relationship between shared IMRGs and immune cells of septic myocardium. We established a promising prognostic model that incorporates *HIF1A* and *SLC25A37* for septic cardiomyopathy. These IMRGs may be potential therapeutic targets for the treatment of septic cardiomyopathy, and small-molecule drugs targeting them may help reduce the sepsis-related death rate.

## Data availability statement

The original contributions presented in the study are included in the article/Supplementary material, further inquiries can be directed to the corresponding authors.

## Ethics statement

The animal study was reviewed and approved by The ethics committee of Shanghai Tenth People’s Hospital, Tongji University.

## Author contributions

RZ carried out the experiments and drafted the articles. RZ, CD, HG, YZ, CL, ZZ, and QW conducted the bioinformatics and statistical analyses. JW and FZ contributed to reviewing the article. SW edited and revised the article. All authors contributed to the article and approved the submitted version.

## Funding

This work was supported by the National Natural Science Foundation of China (No. 81871540) and the Clinical Research Plan of Shanghai Hospital Development Center Foundation (Nos. SHDC2020CR6030-003, SHDC2020CR1028B, and SHDC12020122).

## Conflict of interest

The authors declare that the research was conducted without any commercial or financial relationships that could be construed as a potential conflict of interest.

## Publisher’s note

All claims expressed in this article are solely those of the authors and do not necessarily represent those of their affiliated organizations, or those of the publisher, the editors and the reviewers. Any product that may be evaluated in this article, or claim that may be made by its manufacturer, is not guaranteed or endorsed by the publisher.

## Footnotes

^1^https://online.visual-paradigm.com/diagrams/features/venn-diagram-tool/

^2^http://string-db.org/, version 11.0.

## References

[1] Duran-Bedolla J Montes de Oca-Sandoval MAM Saldaña-Navor V Villalobos-Silva JA Rodriguez MC Rivas-Arancibia S. Sepsis, mitochondrial failure and multiple organ dysfunction. Clin Invest Med. (2014) 37:E58–69. doi: 10.25011/cim.v37i2.21087 24690420

[2] Beesley SJ Weber G Sarge T Nikravan S Grissom CK Lanspa MJ. Septic cardiomyopathy. Crit Care Med. (2018) 46:625–34. doi: 10.1097/CCM.0000000000002851 29227368

[3] Blanco J Muriel-Bombín A Sagredo V Taboada F Gandía F Tamayo L. Incidence, organ dysfunction and mortality in severe sepsis: a Spanish multicentre study. Crit Care. (2008) 12:R158. doi: 10.1186/cc7157, PMID: 19091069 PMC2646323

[4] Karakike E Giamarellos-Bourboulis EJ. Macrophage activation-like syndrome: a distinct entity leading to early death in sepsis. Front Immunol. (2019) 10:55. doi: 10.3389/fimmu.2019.00055, PMID: 30766533 PMC6365431

[5] Su LJ Zhang JH Gomez H Murugan R Hong X Xu D. Reactive oxygen species-induced lipid peroxidation in apoptosis, autophagy, and ferroptosis. Oxidative Med Cell Longev. (2019) 2019:5080843. doi: 10.1155/2019/5080843, PMID: 31737171 PMC6815535

[6] Jankowska EA Kasztura M Sokolski M Bronisz M Nawrocka S Oleśkowska-Florek W. Iron deficiency defined as depleted iron stores accompanied by unmet cellular iron requirements identifies patients at the highest risk of death after an episode of acute heart failure. Eur Heart J. (2014) 35:2468–76. doi: 10.1093/eurheartj/ehu235, PMID: 24927731

[7] Díez-López C Comín-Colet J González-Costello J. Iron overload cardiomyopathy: from diagnosis to management. Curr Opin Cardiol. (2018) 33:334–40. doi: 10.1097/HCO.0000000000000511 29543671

[8] Fang X Ardehali H Min J Wang F. The molecular and metabolic landscape of iron and ferroptosis in cardiovascular disease. Nat Rev Cardiol. (2023) 20:7–23. doi: 10.1038/s41569-022-00735-4, PMID: 35788564 PMC9252571

[9] Zhang H Zhabyeyev P Wang S Oudit GY. Role of iron metabolism in heart failure: from iron deficiency to iron overload. Biochim Biophys Acta Mol basis Dis. (2019) 1865:1925–37. doi: 10.1016/j.bbadis.2018.08.030, PMID: 31109456

[10] Li N Wang W Zhou H Wu Q Duan M Liu C. Ferritinophagy-mediated ferroptosis is involved in sepsis-induced cardiac injury. Free Radic Biol Med. (2020) 160:303–18. doi: 10.1016/j.freeradbiomed.2020.08.009, PMID: 32846217

[11] Xiao Z Kong B Fang J Qin T Dai C Shuai W. Ferrostatin-1 alleviates lipopolysaccharide-induced cardiac dysfunction. Bioengineered. (2021) 12:9367–76. doi: 10.1080/21655979.2021.2001913, PMID: 34787054 PMC8809987

[12] Stevenson EK Rubenstein AR Radin GT Wiener RS Walkey AJ. Two decades of mortality trends among patients with severe sepsis: a comparative meta-analysis*. Crit Care Med. (2014) 42:625–31. doi: 10.1097/CCM.0000000000000026, PMID: 24201173 PMC4313930

[13] Matkovich SJ Al Khiami B Efimov IR Evans S Vader J Jain A. Widespread down-regulation of cardiac mitochondrial and sarcomeric genes in patients with sepsis. Crit Care Med. (2017) 45:407–14. doi: 10.1097/ccm.0000000000002207, PMID: 28067713 PMC5315660

[14] Shalova IN Lim JY Chittezhath M Zinkernagel AS Beasley F Hernández-Jiménez E. Human monocytes undergo functional re-programming during sepsis mediated by hypoxia-inducible factor-1α. Immunity. (2015) 42:484–98. doi: 10.1016/j.immuni.2015.02.001, PMID: 25746953

[15] Gautier L Cope L Bolstad BM Irizarry RA. affy—analysis of Affymetrix GeneChip data at the probe level. Bioinformatics. (2004) 20:307–15. doi: 10.1093/bioinformatics/btg405, PMID: 14960456

[16] Ritchie ME Phipson B Wu D Hu Y Law CW Shi W. limma powers differential expression analyses for RNA-sequencing and microarray studies. Nucleic Acids Res. (2015) 43:e47. doi: 10.1093/nar/gkv007, PMID: 25605792 PMC4402510

[17] Liberzon A Birger C Thorvaldsdóttir H Ghandi M Mesirov JP Tamayo P. The molecular signatures database (MsigDB) hallmark gene set collection. Cell Syst. (2015) 1:417–25. doi: 10.1016/j.cels.2015.12.004, PMID: 26771021 PMC4707969

[18] Ito K Murphy D. Application of ggplot2 to pharmacometric graphics. CPT Pharmacometrics Syst Pharmacol. (2013) 2:e79. doi: 10.1038/psp.2013.56, PMID: 24132163 PMC3817376

[19] Yu G Wang LG Han Y He QY. clusterProfiler: an R package for comparing biological themes among gene clusters. OMICS. (2012) 16:284–7. doi: 10.1089/omi.2011.0118, PMID: 22455463 PMC3339379

[20] Altermann E Klaenhammer TR. PathwayVoyager: pathway mapping using the Kyoto Encyclopedia of Genes and Genomes (KEGG) database. BMC Genomics. (2005) 6:60. doi: 10.1186/1471-2164-6-60, PMID: 15869710 PMC1112592

[21] Szklarczyk D Franceschini A Wyder S Forslund K Heller D Huerta-Cepas J. STRING v10: protein-protein interaction networks, integrated over the tree of life. Nucleic Acids Res. (2015) 43:D447–52. doi: 10.1093/nar/gku1003, PMID: 25352553 PMC4383874

[22] Bader GD Hogue CW. An automated method for finding molecular complexes in large protein interaction networks. BMC Bioinformatics. (2003) 4:2. doi: 10.1186/1471-2105-4-2, PMID: 12525261 PMC149346

[23] Chin CH Chen SH Wu HH Ho CW Ko MT Lin CY. Cytohubba: identifying hub objects and sub-networks from complex interactome. BMC Syst Biol. (2014) 8:S11. doi: 10.1186/1752-0509-8-S4-S11, PMID: 25521941 PMC4290687

[24] Sergushichev A. An algorithm for fast preranked gene set enrichment analysis using cumulative statistic calculation. BioRxiv. (2016) 60012:1–9. doi: 10.1101/060012

[25] Efron B. Logistic regression, survival analysis, and the Kaplan-Meier curve. J Am Stat Assoc. (1988) 83:414–25. doi: 10.1080/01621459.1988.10478612

[26] Yoo M Shin J Kim J Ryall KA Lee K Lee S. DSigDB: drug signatures database for gene set analysis. Bioinformatics. (2015) 31:3069–71. doi: 10.1093/bioinformatics/btv313, PMID: 25990557 PMC4668778

[27] L’Heureux M Sternberg M Brath L Turlington J Kashiouris MG. Sepsis-induced cardiomyopathy: a comprehensive review. Curr Cardiol Rep. (2020) 22:35. doi: 10.1007/s11886-020-01277-2, PMID: 32377972 PMC7222131

[28] Bajpai G Bredemeyer A Li W Zaitsev K Koenig AL Lokshina I. Tissue resident CCR2- and CCR2+ cardiac macrophages differentially orchestrate monocyte recruitment and fate specification following myocardial injury. Circ Res. (2019) 124:263–78. doi: 10.1161/CIRCRESAHA.118.314028, PMID: 30582448 PMC6626616

[29] Epelman S Lavine KJ Beaudin AE Sojka DK Carrero JA Calderon B. Embryonic and adult-derived resident cardiac macrophages are maintained through distinct mechanisms at steady state and during inflammation. Immunity. (2014) 40:91–104. doi: 10.1016/j.immuni.2013.11.019, PMID: 24439267 PMC3923301

[30] Wynn TA Vannella KM. Macrophages in tissue repair, regeneration, and fibrosis. Immunity. (2016) 44:450–62. doi: 10.1016/j.immuni.2016.02.015, PMID: 26982353 PMC4794754

[31] van der Poll T van de Veerdonk FL Scicluna BP Netea MG. The immunopathology of sepsis and potential therapeutic targets. Nat Rev Immunol. (2017) 17:407–20. doi: 10.1038/nri.2017.36, PMID: 28436424

[32] Effenberger-Neidnicht K Hartmann M. Mechanisms of hemolysis during sepsis. Inflammation. (2018) 41:1569–81. doi: 10.1007/s10753-018-0810-y 29956069

[33] Evans C Orf K Horvath E Levin M De La Fuente J Chakravorty S. Impairment of neutrophil oxidative burst in children with sickle cell disease is associated with heme oxygenase-1. Haematologica. (2015) 100:1508–16. doi: 10.3324/haematol.2015.128777, PMID: 26315932 PMC4666326

[34] Zhong H Yazdanbakhsh K. Hemolysis and immune regulation. Curr Opin Hematol. (2018) 25:177–82. doi: 10.1097/MOH.0000000000000423, PMID: 29461260 PMC6309361

[35] Armitage AE Eddowes LA Gileadi U Cole S Spottiswoode N Selvakumar TA. Hepcidin regulation by innate immune and infectious stimuli. Blood. (2011) 118:4129–39. doi: 10.1182/blood-2011-04-351957, PMID: 21873546

[36] Schaefer B Haschka D Finkenstedt A Petersen BS Theurl I Henninger B. Impaired hepcidin expression in alpha-1-antitrypsin deficiency associated with iron overload and progressive liver disease. Hum Mol Genet. (2015) 24:6254–63. doi: 10.1093/hmg/ddv348, PMID: 26310624 PMC4599680

[37] Besson-Fournier C Latour C Kautz L Bertrand J Ganz T Roth MP. Induction of activin B by inflammatory stimuli up-regulates expression of the iron-regulatory peptide hepcidin through Smad1/5/8 signaling. Blood. (2012) 120:431–9. doi: 10.1182/blood-2012-02-411470, PMID: 22611157

[38] Lee P Peng H Gelbart T Wang L Beutler E. Regulation of hepcidin transcription by interleukin-1 and interleukin-6. Proc Natl Acad Sci U S A. (2005) 102:1906–10. doi: 10.1073/pnas.0409808102, PMID: 15684062 PMC548537

[39] Lakhal-Littleton S Wolna M Chung YJ Christian HC Heather LC Brescia M. An essential cell-autonomous role for hepcidin in cardiac iron homeostasis. elife. (2016) 5:e19804. doi: 10.7554/eLife.19804, PMID: 27897970 PMC5176354

[40] Martínez-García JJ Martínez-Banaclocha H Angosto-Bazarra D de Torre-Minguela C Baroja-Mazo A Alarcón-Vila C. P2X7 receptor induces mitochondrial failure in monocytes and compromises NLRP3 inflammasome activation during sepsis. Nat Commun. (2019) 10:2711. doi: 10.1038/s41467-019-10626-x, PMID: 31221993 PMC6586640

[41] Li C Zhang Y Cheng X Yuan H Zhu S Liu J. PINK1 and PARK2 suppress pancreatic tumorigenesis through control of mitochondrial iron-mediated immunometabolism. Dev Cell. (2018) 46:441–455.e8. doi: 10.1016/j.devcel.2018.07.012, PMID: 30100261 PMC7654182

[42] Angus DC van der Poll T. Severe sepsis and septic shock. N Engl J Med. (2013) 369:840–51. doi: 10.1056/NEJMra1208623 23984731

[43] Islam S Jarosch S Zhou J Parquet Mdel C Toguri JT Colp P. Anti-inflammatory and anti-bacterial effects of iron chelation in experimental sepsis. J Surg Res. (2016) 200:266–73. doi: 10.1016/j.jss.2015.07.001, PMID: 26235905

[44] Xia Y Farah N Maxan A Zhou J Lehmann C. Therapeutic iron restriction in sepsis. Med Hypotheses. (2016) 89:37–9. doi: 10.1016/j.mehy.2016.01.018, PMID: 26968906

[45] Goto M Suematsu Y Nunes ACF Jing W Khazaeli M Lau WL. Ferric citrate attenuates cardiac hypertrophy and fibrosis in a rat model of chronic kidney disease. Iran J Kidney Dis. (2019) 13:98–104. PMID: 30988246

[46] Ito K Hirooka Y Sunagawa K. Cardiac sympathetic afferent stimulation induces salt-sensitive sympathoexcitation through hypothalamic epithelial Na+ channel activation. Am J Physiol Heart Circ Physiol. (2015) 308:H530–9. doi: 10.1152/ajpheart.00586.2014, PMID: 25527778

[47] Ichikawa Y Ghanefar M Bayeva M Wu R Khechaduri A Naga Prasad SVN. Cardiotoxicity of doxorubicin is mediated through mitochondrial iron accumulation. J Clin Invest. (2014) 124:617–30. doi: 10.1172/JCI72931, PMID: 24382354 PMC3904631

[48] Tucker A. Pulmonary vascular actions of the antihistamine oxatomide during hypoxia. Agents Actions. (1980) 10:207–12. doi: 10.1007/BF02025937, PMID: 6157318

[49] Singer M Deutschman CS Seymour CW Shankar-Hari M Annane D Bauer M. The third international consensus definitions for sepsis and septic shock (Sepsis-3). JAMA. (2016) 315:801–10. doi: 10.1001/jama.2016.0287, PMID: 26903338 PMC4968574

