# Supplementary material for: Identification of iron metabolism-related genes in the circulation and myocardium of patients with sepsis via applied bioinformatics analysis
Source: Front Cardiovasc Med. 2023 Mar 2;10:1018422. doi: 10.3389/fcvm.2023.1018422 (PMC10017502; doi:10.3389/fcvm.2023.1018422)
Supplement: Supplementary file 1 [file Data_Sheet_1.PDF]

Supplementary Material

1 Supplementary Figures and Tables

Supplementary Table 1: Primes sequence.

Genes	Forward	Reverse
SLC25A37	CCTACTCCACGATGCAGTAATG	AGTGAATTGACTGGAAGGGGATA
HIF1A	ACCTTCATCGGAAACTCCAAAG	CTGTTAGGCTGGGAAAAGTTAGG
β-actin	GGCTGTATTCCCCTCCATCG	CCAGTTGGTAACAATGCCATGT

Supplementary Table 2: The score of hub genes identified by Cytohubba.

Rank	Name	Score
1	HAMP	10
2	SLC25A37	9
3	SLC39A14	7
3	SLC11A1	7
5	HMOX1	5
6	SOD2	4
7	HIF1A	4
8	GLRX5	2
8	HBB	2
10	NCOA4	1

Supplementary Table 3: Multivariate Cox regression analysis.

Genes	coef	HR	HR.95L	HR.95H	pvalue
HIF1A	0.42386721	1.5278587	1.16411599	2.00525738	0.00224801
SLC25A37	0.88143471	2.41436114	1.37152234	4.25012379	0.00225144

Supplementary Table 4: The expression levels (LogFC) of key genes with values from microarray and qPCR.

Genes	Microarray	qPCR
HIF1A	1.53192753	1.67109012
SLC25A37	1.47315093718634	3.19291652

2 Supplementary Figures

Supplementary Figure 1: The forest plot of the Multivariate Cox regression for independent prognostic analysis

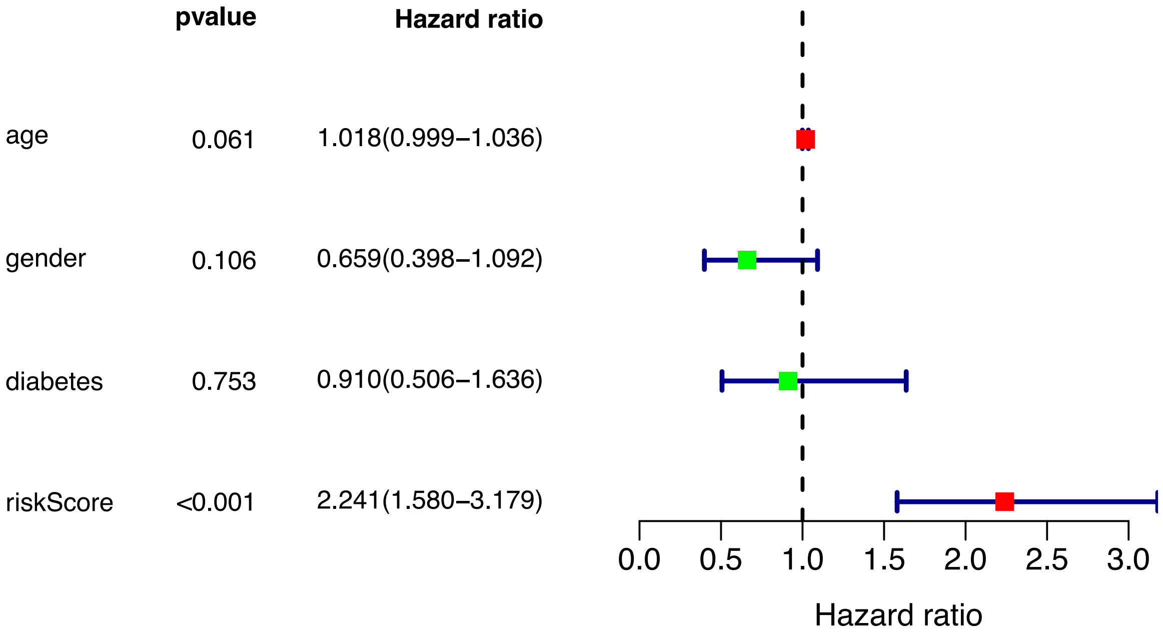
pvalue
Hazard ratio
age
0.061
1.018(0.999−1.036)
gender
0.106
0.659(0.398−1.092)
diabetes
0.753
0.910(0.506−1.636)
riskScore
<0.001
2.241(1.580−3.179)
0.0
0.5
1.0
1.5
2.0
2.5
3.0
Hazard ratio
